# Additions to tribe Chromosereae (Basidiomycota, Hygrophoraceae) from China, including *Sinohygrocybe* gen. nov. and a first report of *Gloioxanthomycesnitidus*

**DOI:** 10.3897/mycokeys.38.25427

**Published:** 2018-08-28

**Authors:** Chao-Qun Wang, Ming Zhang, Tai-Hui Li, Xi-Shen Liang, Ya-Heng Shen

**Affiliations:** 1 State Key Laboratory of Applied Microbiology Southern China, Guangdong Provincial Key Laboratory of Microbial Culture Collection and Application, Guangdong Open Laboratory of Applied Microbiology, Guangdong Institute of Microbiology, Guangzhou 510070, China Guangdong Institute of Microbiology Guangzhou China

**Keywords:** East Asia, new record species, new taxa, phylogeny overview

## Abstract

*Sinohygrocybe***gen. nov.**, typified by *S.tomentosipes***sp. nov.**, is described upon morphological and molecular evidence. The new genus is characterised by its sinuate to subdecurrent or short deccurent, usually furcate and interveined and relatively distant lamellae, dry and whitish tomentose stipe, thin-walled ellipsoid to oviod, non-constricted basidiospores and particularly elongated basidia and a ratio of basidiospore to basidium length of >5 to 8; it is close to genera *Chromosera* and *Gloioxanthomyces* of the tribe Chromosereae, but morphologically differs from *Chromosera* in less umbilicate basidiomata, tomentose stipe and usually longer basidia and differs from *Gloioxanthomyces* in more robust basidioma and less glutinous pileus and/or stipe surface. Phylogenetic analyses, with ITS-LSU-RPB2 data, also indicate that *Sinohygrocybe* forms a very distinct and independent clade at the generic level. In addition, a Chinese new record *G.nitidus* is described here.

## Introduction

Hygrophoraceae Lotsy (Hymenomycetes, Basidiomycota) is a large family in Agaricales, including 26 genera and over 600 species (Lodge et al. 2014). In a six-gene phylogenetic tree of Agaricales, Hygrophoraceae, Pterulaceae Corner, Typhulaceae Jülich and some small groups formed a Hygrophoroid clade, which is one of the six largest clades in Agaricales (Matheny et al. 2006); and in a genome based mushroom tree of life, Hygrophoraceae and Clavariaceae Chevall. are representative families of the suborder Hygrophorineae Aime, Dentinger & Gaya, which is one of the seven suborders of the Agaricales (Dentinger et al. 2016). Traditionally, the family Hygrophoraceae referred to a group of agaricoid, waxy-gilled and white-spored mushrooms; and a majority of the members are classified in the type genus *Hygrophorus* Fr. and genus *Hygrocybe* (Fr.) P. Kumm. Morphological characters of the Hygrophoraceae taxa are relatively simple (usually without annulus or volva and a cystidiate) amongst the agaric fungi and their basidioma colours are often very susceptible to the environmental conditions and developmental stages, making their classification and identification difficult, so it is often challenging to make correct identification and taxonomy of them just according to morphological recognition (Young 2005). Modern molecular techniques have been revolutionising the taxonomy and phylogeny of Hygrophoraceae.

Lodge et al. (2014) had conducted the most comprehensive molecular phylogenetic study on the family until now, therefore their systematic viewpoint on Hygrophoraceae is adopted in this paper. According to their study, the family could be divided into four groups at subfamily level, i.e. subfamily Hygrophoroideae E. Larss., Lodge, Vizzini, Norvell & S.A. Redhead, Hygrocyboideae Padamsee & Lodge, Lichenomphalioideae Lücking & Redhead and Cuphophylloid grade. The subfamily Hygrocyboideae could be divided into three tribes, i.e. tribe Chromosereae, Humidicuteae and Hygrocybeae; and the tribe Chromosereae included two sister genera, *Chromosera* Redhead, Ammirati & Norvell and *Gloioxanthomyces* Lodge, Vizzini, Ercole & Boertm.

*Chromosera*, the type genus of the tribe Chromosereae, was erected to accommodate *Omphalinacyanophylla* (Fr.) Quél. which was originally described from Sweden and combined as *C.cyanophylla* (Fr.) Redhead, Ammirati & Norvell (Redhead et al. 1995, 2012). Now, another four species, formerly placed in *Hygrocybe* or *Hygrophorus*, are also classified into *Chromosera*, i.e. *C.citrinopallida* (A.H. Sm. & Hesler) Vizzini & Ercole originally described from USA, *C.lilacina* (P. Karst.) Vizzini & Ercole originally described from the northern Fennoscandia, *C.viola* (J. Geesink & Bas) Vizzini & Ercole originally described from Belgium and *C.xanthochroa* (P.D. Orton) Vizzini & Ercole originally described from Scotland (Lodge et al. 2014).

*Gloioxanthomyces* is a small genus with only two known species, the type species *G.vitellinus* (Fr.) Lodge, Vizzini, Ercole & Boertm. originally described from Europe and *G.nitidus* (Berk. & M.A. Curtis) Lodge, Vizzini, Ercole & Boertm. from North America (Crous et al. 2004, Lodge et al. 2014). Before the recognition of *Gloioxanthomyces*, those two species were usually placed in the genus *Hygrocybe* as *H.vitellina* (Fr.) P. Karst and *H.nitida* (Berk. & M.A. Curtis) Murrill, respectively. Morphologically, the main differences between the two species were in their basidiospore sizes: *G.nitidus* had ellipsoid to oblong basidiospores, measuring 7–10 × 5–6 μm with Q = 1.3–1.8; while *G.vitellinus* had subglobose basidiospores, measuring 6.5–8.5 × 5–7 μm with Q=1.1–1.6 (Boertmann 1990). Since their differences were limited, the two taxa seemed to be conspecific (Boertmann 2011). However, according to the phylogenetic analyses with ITS data by Boertmann (2012), the European collections clearly clustered together as the *G.vitellinus* species clade, while the North American materials independently formed another group as the *G.nitidus* species clade, thus they could actually be sharply defined as two separated sister species.

During the studies on the Chinese Hygrophoraceae in recent years, some collections morphologically corresponding to tribe Chromosereae were collected. Comprehensive observation and analyses revealed some interesting findings, which can contribute to the taxonomic knowledge of the tribe. In this paper, we aim to: 1) formally describe a new genus of tribe Chromosereae from East Asia based upon morphological and molecular analyses and present a Chinese new record of *Gloioxanthomycesnitidus*; 2) reconstruct the phylogeny of the family Hygrophoraceae using 3 gene regions, i.e. the internal transcribed spacer region (ITS), the large subunit nuclear ribosomal RNA region (nrLSU) and the nuclear RPB2 6F to 7.1R region (RPB2). Detailed studies were therefore conducted and the results are presented as follows.

## Materials and methods

### Morphological studies

Specimens were photographed and annotated in the field and then dried in an electric drier. Macroscopic descriptions were gained from the original field notes and photographs. Colour descriptions followed Kornerup and Wanscher (1978). Tissue sections were immersed in 5% potassium hydroxide (KOH) and/or 1% Congo Red solution for microscopical examinations, but in distilled water for colour descriptions of basidia, pileipellis and stipitipellis. From a mature specimen, over 40 basidiospores and 20 basidia were randomly selected and measured under a light microscope in KOH. The notation (a)b–c(d) was used to describe dimensions where the range b–c representing 90% or more of the measured values and a, d were the extreme values. The length/width ratio of spores was presented as Q and the mean ratio was presented as Q_m_. The studied specimens were deposited in the Fungal Herbarium of Guangdong Institute of Microbiology (GDGM), Guangzhou, China.

### Molecular studies

Genomic DNA was extracted from the herbarium specimens using the Sangon Fungus Genomic DNA Extraction kit (Sangon Biotech Co., Ltd., Shanghai, China) according to the manufacturer’s instructions. The ITS, LSU and RPB2 gene regions were amplified by Polymerase Chain Reaction, using universal primers ITS1F/ITS5 and ITS4 (White et al. 1990; Gardes and Bruns 1993), LR0R and LR5 (http://biology.duke.edu/fungi/mycolab/primers.htm) and RPB2-6F and RPB2-7.1R (Matheny 2005), respectively. Amplified products were sequenced by Beijing Genomic Institute (BGI) using the same primers. The abi format sequences were assembled by SeqMan version 7.1.0 (DNAStar, Inc.) and then the assembled sequences were submitted to GenBank.

In this study, two datasets were constructed. The first one is an ITS-LSU-RPB2 matrix of the family Hygrophorceaeae for making a comprehensive phylogenetic tree and analysing the positions of the new taxa; most known species of Hygrophoraceae with available sequences from reliable sources were included in the dataset, each of them having at least an LSU sequence and *Typhulaphacorrhiza* (Reichard) Fr. was selected as the outgroup referred from Yang et al. (2013) and Lodge et al. (2014). The second dataset is an ITS matrix of the tribe Chromosereae and *Hygrocybeconica* (Schaeff.) P. Kumm. and H.conicavar.conicoides (P.D. Orton) Boertm. were chosen as outgroups. Each gene was independently aligned on the online MAFFT service (Katoh et al. 2017), then combined by the Geneious software (Biomatters Ltd.) for the first dataset. Maximum likelihood phylogenetic trees were generated by the RAxML software (Stamatakis 2014) on the CIPRES service (Miller et al. 2010) with 1000 bootstrap replications using the default options.

## Results

### Molecular phylogenetic results

**Figure 1.**
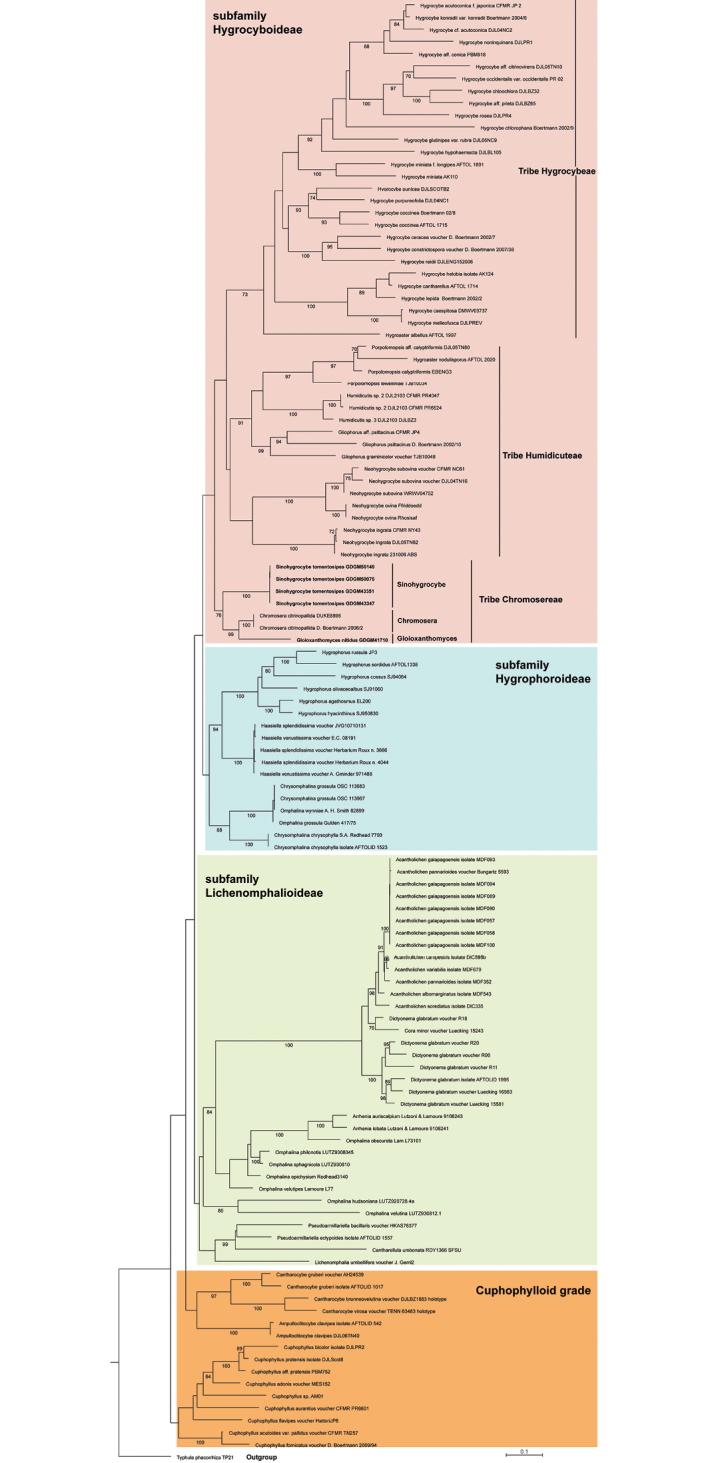
Phylogenetic overview of the family Hygrophoraceae inferred from ITS-LSU-RPB2 data using Maximum Likelihood (ML) method. *Typhulaphacorrhiza* was selected as outgroup. Bootstrap values (≥50%) were presented around the branches. The newly generated sequences are shown in bold.

The combined 3-gene dataset composed of 120 samples (Table 1), including 5 newly sequenced samples and 115 published ones. In the final matrix, the ITS, LSU and RPB2 regions comprised positions 1 to 1751, 1752 to 2873, 2874 to 3759, respectively. In the 3-gene Maximum Likelihood tree (Fig. 1), the four Chinese collections (GDGM43351 and GDGM43347 from Sichuan province, GDGM50075 and GDGM50149 from Hunan province) formed a strong monophyletic clade with 100% bootstrap support, which was near the *Chromosera*-*Gloioxanthomyces* clade composed of members of *Chromosera* and *Gloioxanthomyces* with 76% bootstrap support.

**Table 1.** Sequences information of samples used for the ITS-LSU-RPB2 combined tree. Newly generated sequences were bold.

Species name	Isolate/voucher ID	ITS	LSU	RPB2
* Acantholichen albomarginatus *	MDF543	KT429797	KT429809	–
* Acantholichen campestris *	DIC595b	KT429798	KT429810	KT429818
* Acantholichen galapagoensis *	MDF057	KT429784	KT429799	KT429811
* Acantholichen galapagoensis *	MDF058	KT429785	KT429800	KT429812
* Acantholichen galapagoensis *	MDF089	KT429786	KT429801	–
* Acantholichen galapagoensis *	MDF090	KT429787	KT429802	KT429813
* Acantholichen galapagoensis *	MDF093	KT429790	KT429803	KT429814
* Acantholichen galapagoensis *	MDF094	KT429791	KT429804	KT429815
* Acantholichen galapagoensis *	MDF100	KT429792	KT429805	KT429816
* Acantholichen pannarioides *	MDF352	KT429795	KT429807	KT429817
* Acantholichen pannarioides *	Bungartz 5593	EU825953	EU825953	–
* Acantholichen sorediatus *	DIC335	KT429794	KT429806	–
* Acantholichen variabilis *	MDF679	KT429796	KT429808	–
* Ampulloclitocybe clavipes *	DJL06TN40	–	KF381542	KF407938
* Ampulloclitocybe clavipes *	AFTOL-ID 542	AY789080	AY639881	AY780937
* Arrhenia auriscalpium *	Lutzoni Lamoure 910824-3	U66428	U66428	–
* Arrhenia lobata *	Lutzoni Lamoure 910824-1	U66429	U66429	–
* Cantharellula umbonata *	RDY-1366 (SFSU)	KF381519	AF261443	–
* Cantharocybe brunneovelutina *	DJL-BZ-1883 (holotype)	KX452404	HM588721	–
* Cantharocybe gruberi *	AFTOL-ID 1017	DQ200927	DQ234540	DQ385879
* Cantharocybe gruberi *	AH24539	JN006422	JN006420	–
* Cantharocybe virosa *	TENN 63483(holotype)	KX452405	JX101471	–
* Chromosera citrinopallida *	DUKE8895	U66435	U66435	–
* Chromosera citrinopallida *	D. Boertmann 2006/2	KF291072	KF291073	–
* Chrysomphalina chrysophylla *	AFTOL-ID 1523	–	DQ457656	DQ192180
* Chrysomphalina chrysophylla *	S.A. Redhead 7700	–	U66430	U66430
* Chrysomphalina grossula *	OSC 113667	–	EU652372	EU644703
* Chrysomphalina grossula *	OSC 113683	–	EU652373	EU644704
* Cora minor *	Luecking 15243	EU825968	EU825968	–
Cuphophyllus acutoides var. pallidus	CFMR TN-257	–	KF291097	–
* Cuphophyllus adonis *	MES-152	–	KF291036	KF291037
Cuphophyllus aff. pratensis	PBM-752	–	DQ457650	KF442252
* Cuphophyllus aurantius *	CFMR PR-6601	–	KF291100	KF291102
* Cuphophyllus bicolor *	DJL-PR-2	–	KF291056	–
* Cuphophyllus flavipes *	Hattori-JP-6	–	KF291045	KF291047
* Cuphophyllus fornicatus *	D. Boertmann 2009/94	–	KF291124	–
* Cuphophyllus pratensis *	DJL-Scot-8	–	KF291058	–
*Cuphophyllus* sp.	AM01	–	HM026542	–
* Dictyonema glabratum *	AFTOL-ID 1995	DQ917656	DQ917661	–
* Dictyonema glabratum *	Luecking 15581	EU825958	EU825958	–
* Dictyonema glabratum *	Luecking 16563	EU825956	EU825956	–
* Dictyonema glabratum *	R06	EU825959	EU825959	–
* Dictyonema glabratum *	R11	EU825960	EU825960	–
* Dictyonema glabratum *	R18	EU825961	EU825961	–
* Dictyonema glabratum *	R20	EU825963	EU825963	–
Gliophorus aff. psittacinus	CFMR JP-4	KF291079	KF291080	–
* Gliophorus graminicolor *	TJB-10048	KF381520	KF381545	KF407936
* Gliophorus psittacinus *	D. Boertmann 2002/10	KF291075	KF291076	KF291078
* Gloioxanthomyces nitidus *	GDGM41710	**MG712283-4**	**MG712282**	**MG711911**
* Haasiella splendidissima *	Herbarium Roux n. 3666	JN944398	JN944399	–
* Haasiella splendidissima *	Herbarium Roux n. 4044	JN944400	JN944401	–
* Haasiella splendidissima *	JVG1071013-1	JN944395	JN944396	–
* Haasiella venustissima *	A. Gminder 971488	KF291092	KF291093	–
* Haasiella venustissima *	E.C. 08191	JN944393	JN944394	–
*Humidicutis* sp. 2	CFMR PR4047	–	KF291151	KF291149
*Humidicutis* sp. 2	DJL-2103 CFMR PR-6524	KF291150	KF291151	
*Humidicutis* sp. 3	D.J. Lodge DJL-BZ-3	KF291110	KF291111	–
* Hygroaster albellus *	AFTOL ID 1997	KF381521	EF551314	KF381510
* Hygroaster nodulisporus *	AFTOL-ID 2020	–	EF561625	KF381511
Hygrocybe acutoconica f. japonica	CFMR JP-2	KF291161	KF291162	
Hygrocybe aff. citrinovirens	DJL05TN10	KF291090	KF291091	–
Hygrocybe aff. conica	PBM 918	AY854074	DQ071739	AY803747
Hygrocybe aff. prieta	DJL-BZ-65	KF291168	KF291169	
* Hygrocybe caespitosa *	DMWV-03-737	KF291104	KF291105	KF291107
* Hygrocybe cantharellus *	AFTOL-ID 1714	DQ490628	DQ457675	
* Hygrocybe ceracea *	D. Boertmann 2002/7	KF291108	KF291109	–
Hygrocybe cf. acutoconica	DJL04NC2	KF291117	KF291118	KF291120
* Hygrocybe chloochlora *	DJL-BZ-32	EU435147	EU435147	–
* Hygrocybe chlorophana *	Boertmann 2002/9	EU435148	EU435148	KF381513
* Hygrocybe coccinea *	AFTOL-ID 1715	DQ490629	DQ457676	DQ472723
* Hygrocybe coccinea *	Boertmann02/8	EU435146	EU435146	KF291114
* Hygrocybe constrictospora *	D. Boertmann 2007/38	KF291115	KF291116	
Hygrocybe glutinipes var. rubra	DJL05NC9	EU435149	EU435149	–
* Hygrocybe helobia *	AK-124	KF291182	KF291183	–
* Hygrocybe hypohaemacta *	DJL-BZ-105	EU435150	EU435150	KF291165
Hygrocybe konradii var. konradii	Boertmann 2004/6	KF306329	KF306330	–
* Hygrocybe lepida *	Boertmann 2002/2	KF306333	KF306334	–
* Hygrocybe melleofusca *	DJL-PR-EV	KF291154	KF291155	–
* Hygrocybe miniata *	AK-110	KF291179	KF291180	
Hygrocybe miniata f. longipes	AFTOL-ID 1891	DQ490630	DQ457677	DQ472724
* Hygrocybe noninquinans *	DJL-PR-1	KF291127	KF291129	KF291128
Hygrocybe occidentalis var. occidentalis	Cancerel PR 02	EU435151	EU435151	–
* Hygrocybe punicea *	DJL-SCOT-B2	KF291133	KF291134	–
* Hygrocybe purpureofolia *	DJL04NC1	KF291192	KF291193	
* Hygrocybe reidii *	DJL-ENG-15-2006	KF291158	KF291159	
* Hygrocybe rosea *	DJL-PR-4	KF291197	KF291198	–
* Hygrophorus agathosmus *	EL2-00	–	AY586660	–
* Hygrophorus cossus *	SJ94064	AY548963	AY548963	
* Hygrophorus hyacinthinus *	SJ950830	–	HM143012	–
* Hygrophorus olivaceoalbus *	SJ91060	–	AY586662	–
* Hygrophorus russula *	JP-3	KF291216	KF291217	KF291219
* Hygrophorus sordidus *	AFTOL-1338	DQ490632	AF042562	–
* Lichenomphalia umbellifera *	J. Geml-2	U66445	U66445	KF381515
* Neohygrocybe ingrata *	GWG H. ingrata 23-10-06 (ABS)	KF291225	KF291226	–
* Neohygrocybe ingrata *	TN-62 voucher DJL05TN62	KF381525	KF381558	KF381516
* Neohygrocybe ingrata *	CFMR NY-43	–	KF291223	KF291224
* Neohygrocybe ovina *	K(M) 187568	KF291228	KF291229	–
* Neohygrocybe ovina *	GWG H. ovina Rhosisaf (ABS)	KF291233	KF291234	KF291236
* Neohygrocybe subovina *	WRWV04-752 (DEWV 5366)	–	KF291142	KF291138
* Neohygrocybe subovina *	CFMR NC-61	KF291136	KF291137	–
* Neohygrocybe subovina *	DJL04TN16 (GRSM 77065)	KF291140	KF291141	–
* Omphalina epichysium *	Redhead3140	U66442	U66442	–
* Omphalina grossula *	Gulden 417/75	–	U66444	U66444
* Omphalina hudsoniana *	LUTZ-920728.4a	U66446	U66446	–
* Omphalina obscurata *	Lam L73-101	U66448	U66448	–
* Omphalina philonotis *	LUTZ930804-5	U66449	U66449	–
* Omphalina sphagnicola *	LUTZ930810	U66453	U66453	–
* Omphalina velutina *	LUTZ-930812.1	U66454	U66454	–
*Omphalina velutipes Lamoure*	L77	U66455	U66455	–
*Omphalinawynniae* A. H. Smith	82899	–	U66457	U66457
Porpolomopsis aff. calyptriformis	DJL05TN80	KF291246	KF291247	KF291249
* Porpolomopsis calyptriformis *	EB-ENG-3	KF291242	KF291243	KF291245
* Porpolomopsis lewelliniae *	TJB-10034	KF291238	KF291239	KF291241
* Pseudoarmillariella bacillaris *	HKAS76377	KC222315	KC222316	–
* Pseudoarmillariella ectypoides *	AFTOL-ID 1557	DQ192175	DQ154111	DQ474127
* Sinohygrocybe tomentosipes *	GDGM43351	**MG685872**	**MG696901**	**MG696905**
* Sinohygrocybe tomentosipes *	GDGM43347	–	**MG696900**	**MG696904**
* Sinohygrocybe tomentosipes *	GDGM50075	**MG685873**	**MG696902**	**MG696906**
* Sinohygrocybe tomentosipes *	GDGM50149	**MG685874**	**MG696903**	**MG675232**
* Typhula phacorrhiza *	TP21	AF134710	AF393079	AY218525

**Figure 2.**
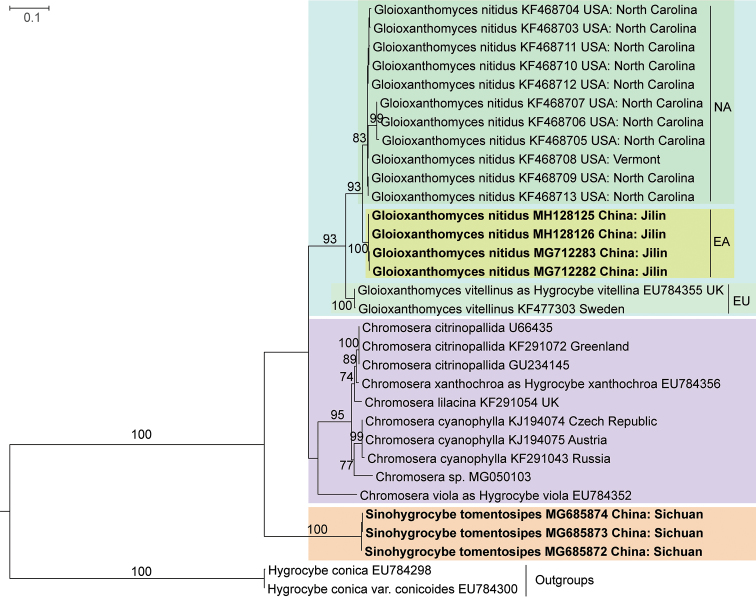
Phylogenetic overview of the tribe Chromosereae inferred from ITS data using ML method. Two *Hygrocybeconica* sequences were rooted as outgroups. Bootstrap values (≥50%) are shown around the branches. GenBank accession numbers of downloaded sequences were added after the species name and the collection locations were added at the ends. NA, EA and EU referred to North America, East Asia and Europe, respectively. The newly generated sequences are shown in bold.

The ITS dataset included 30 samples of all known taxa of tribe Chromosereae and 2 *Hygrocybe* sequences chosen as the outgroups, the matrix length is 679 bp. In the ITS Maximum Likelihood tree (Fig. 2), collections of the species *G.nitidus* and *G.vitellinus* were clustered together with 93% and 100% support values, respectively and the North American and the East Asian *G.nitidus* were clustered as sister groups with 93% support value; all the members of *Chromosera* (except *C.viola*), *Gloioxanthomyces* and *Sinohygrocybe* were clustered together with 95%, 93% and 100% support values, respectively; and the *Chromosera*-*Gloioxanthomyces* clade was presented as the sister clade of the *Sinohygrocybe* clade with strong support value (100%).

### Taxonomy

#### Sinohygrocybe

Taxon classificationFungiAgaricalesHygrophoraceae

C.Q. Wang, Ming Zhang & T.H. Li gen. nov.

MB824821

##### Diagnosis.

Differs from *Chromosera* and *Gloioxanthomyces* by its less omphalioid, more robust basidiomata, dry to subviscid pileus, dry and white tomentose stipe, more elongated basidia, higher length ratio (up to 8 times) of basidia to basidospores.

##### Etymology.

*Sino*- refers China, the holotype’s location of the genus; -*hygrocybe* indicates that it is a *Hygrocybe*-like genus.

##### Type species.

*Sinohygrocybetomentosipes* C.Q. Wang, Ming Zhang & T.H. Li

##### Description.

Basidiomata medium-sized, subcaespiotose. Pileus convex to applanate, slightly depressed in the centre, yellow, orangish-yellow to orange, dry to subviscid, slightly when wet, never strongly gelatinised or glutinous. Lamellae adnate to decurrent, concolorous with pileus, with usually furcate and interveined lamellulae. Stipe yellow to whitish or almost concolorous with pileus, yellow or covered by white to yellowish-white tomentum. Basidiospores ellipsoid to oblong, ovoid, Qm = 1.6-1.7, not constricted, thin-walled, inamyloid, hyaline, smooth; basidia usually 4-sterigmate, 41–80 μm long, ratio of basidia to basidiospore length over 5 (up to 8), with basal clamp connection. Pileipellis and stipitipellis a cutis. Lamellar trama subregular. Clamp connections present throughout.

#### Sinohygrocybe tomentosipes

Taxon classificationFungiAgaricalesHygrophoraceae

C.Q. Wang, Ming Zhang & T.H. Li sp. nov.

MB824824

Figs 3 , 4

##### Diagnosis.

Differs from the other members of the tribe Chromosereae by its larger and more robust basidiomata, concolorous yellow pileus, lamellae and the subsurface of stipe, usually furcate and interveined lamellae and lamellulae, white fibrillose stipe surface, long basidia (up to 80 μm), ratio of basidia to basidiospore length over 5 and even up to 8.

##### Etymology.

The species epithet *tomentosipes* refers to the tomentose stipe.

##### Type.

China. Sichuan Province, Panzhihua City, Yanbian County, Gesala Eco-tourism Area, at 27°16'N, 101°26'E, alt. 3100 m, 24 Aug 2013, Ming Zhang (GDGM43351, holotype).

##### Description.

Basidiomata small to medium-sized. Pileus 2.5–6 cm diam., convex to applanate, usually slightly depressed in the centre, smooth, dry but subviscid when wet, light yellow to vivid yellow (3A5–8) or to deep yellow (4A5–8), or light orange to dark orange (5A5–8), becoming paler when dry; margin even, straight or upturned and occasionally split when mature. Lamellae up to 7 mm wide, adnate to sinuate or decurrent, distant, 17–22 lamellae per pileus, with 1–3 lamellulae between two complete lamellae, usually furcate, often interveined or anastomosing at lamella base, thick, concolorous with the pileus; lamellar base and lamellulae irregular and occasionally the whole hymenophore irregular; lamellar edge even and concolorous. Context concolorous with lamellae and pileus, unchanged when cut. Stipe 4–6.5 × 0.6–1.2 cm, central or occasionally eccentric, subcylindrical, moderately to densely covered with white tiny adpressed fibres. Odour indistinct.

Basidiospores 8–10(–10.5) × (4.5–)5–7(–7.5) μm, Q = (1.3–)1.5–1.8, Q_m_ = 1.6–1.7, ellipsoid to ellipsoid-oblong, ovoid, not constricted, thin-walled, hyaline, smooth. Basidia 41–80 × 4–10 μm, strongly elongated, narrow clavate, 4-spored, thin-walled; sterigmata up to 10 μm long; ratio of basidia to basidiospore length over 5 and up to 8. Hymenophoral trama subregular, yellow, made up of thin-walled hyphae 3–15 µm wide and usually less than 100 μm long and some conducting elements. Pileipellis a cutis, made up of repent hyphae 3–9 µm wide with the terminal elements 30–80 µm long. Stipitipellis a cutis, with thin-walled hyphae (5–7 μm wide). Clamp-connections present in all tissues.

##### Habitat and known distribution.

Gregarious, caespitose, or scattered in broad-leaf forest in subtropical temperate transition zone, so far known only from Sichuan and Hunan Provinces in China.

##### Additional specimens examined.

CHINA, Sichuan Province, Panzhihua City, Yanbian County, Gesala Eco-Tourism Area, at 27°16'N, 101°26'E, alt. 3100 m, 24 Aug 2013, Ming Zhang (GDGM43347), Chao-Qun Wang (GDGM43352); Hunan Province, Zhuzhou City, Yanling County, Taoyuandong National Nature Reserve, at 26°19'N, 114°00'E, alt. 1534 m, 23 Nov 2013, Chao-Qun Wang (GDGM50075 and GDGM50149).

**Figure 3.**
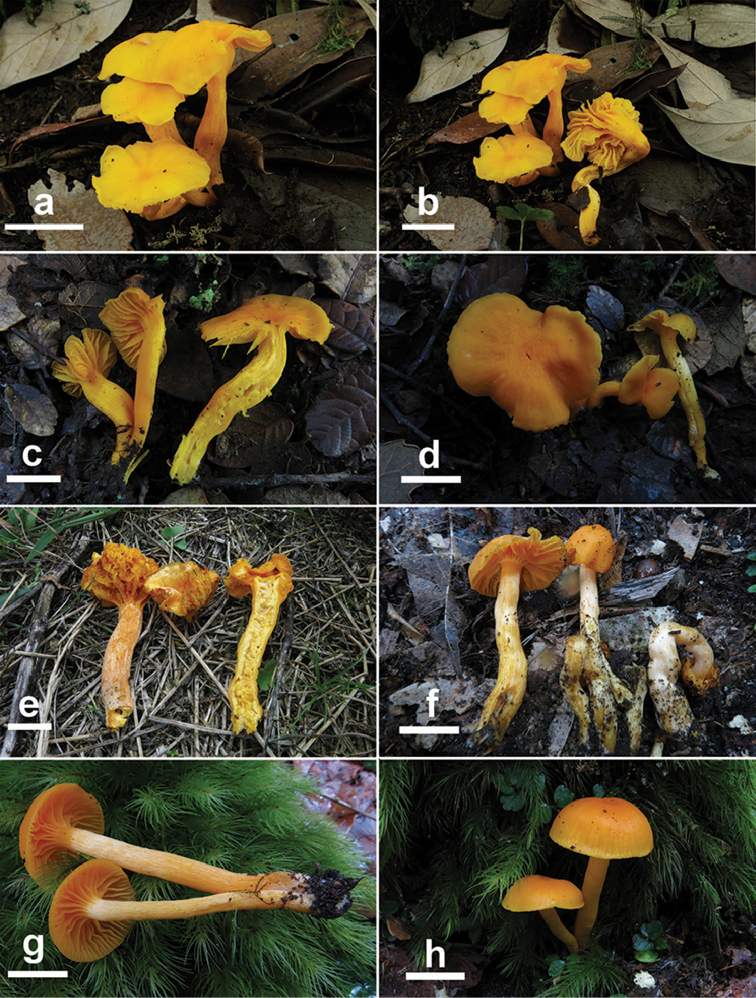
Basidiomata of *Sinohygrocybetomentosipes* (**a–b** GDGM43351 **c–d** GDGM43352 **e** GDGM43347 **f** GDGM50075 **g–h** GDGM50149). Scale bars: 2 cm.

**Figure 4.**
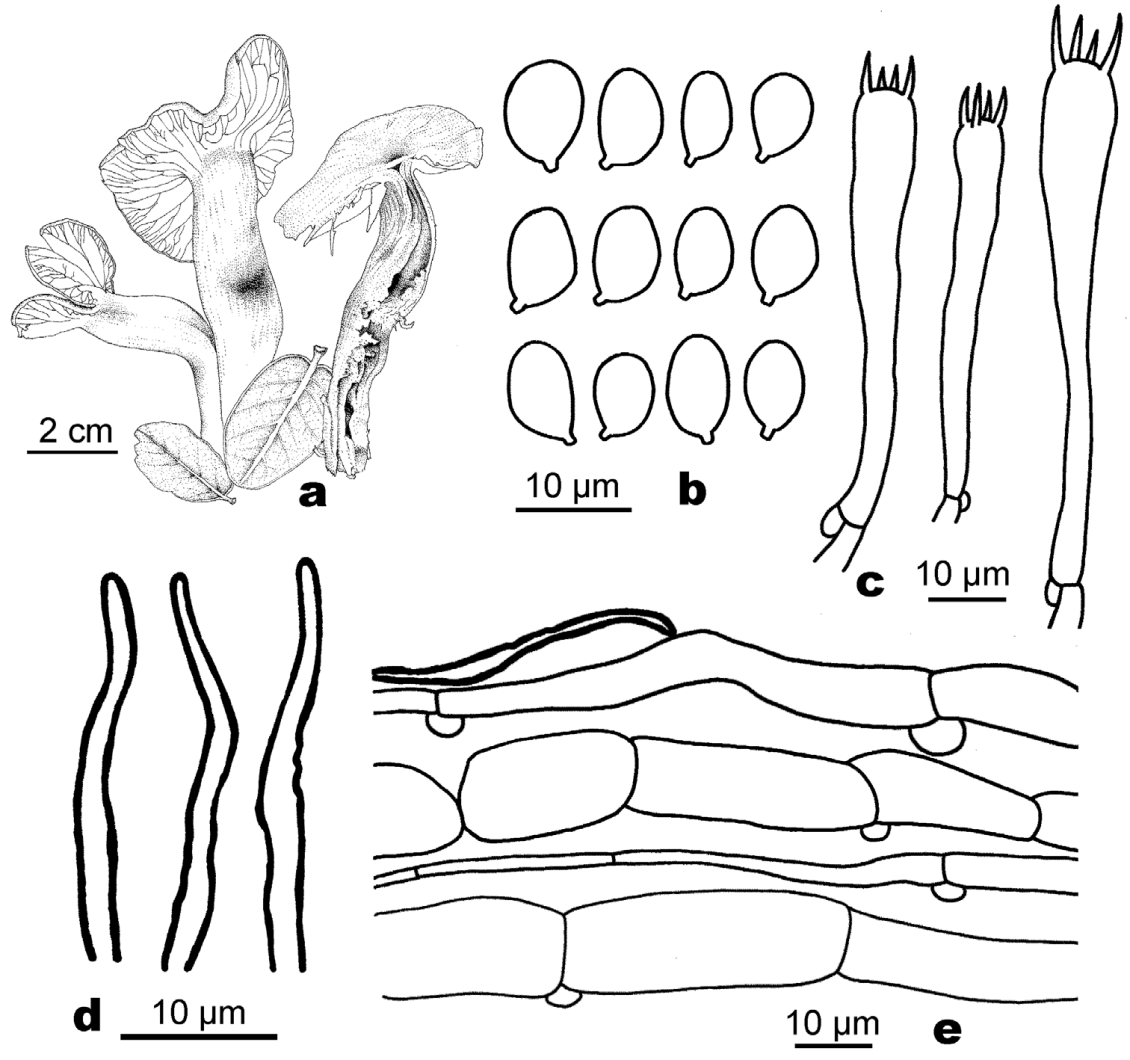
Line drawings of *Sinohygrocybetomentosipes*. **a** Basidiomata **b** Basidiospores **c** Basidia **d** Elements of pileipellis cells **e** Elements of gill trama.

#### Gloioxanthomyces nitidus

Taxon classificationFungiAgaricalesHygrophoraceae

(Berk. & M.A. Curtis) Lodge, Vizzini, Ercole & Boertm., Fungal Diversity 64: 50 (2014)

Figs 5 , 6

= Hygrophorusnitidus Berk. & M.A. Curtis, Ann. Mag. nat. Hist., Ser. 2 12: 424 (1853).

##### Description.

Pileus 1.5–3.5 cm wide, convex to nearly plane with a slightly depressed disc, strongly glutinous, yellow, light orange yellow to apricot yellow, even whitish-yellow when mature, clearly striate at margin; pileus margin usually slightly undulating, slightly incurved when young, expanded to flat or partially uplifted when mature. Context thin, yellow to nearly concolorous with pileus, hygrophanous and translucent. Lamellae arcuate-decurrent, narrow at both ends, bright yellow or slightly orange yellow, waxy and fragile, subdistant, usually having 1–3 unequal lamellulae between two lamellae; lamellar edge even, usually gelatinised and sometimes translucent. Stipe 2.5–6 × 0.2–0.5 cm, cylindrical, hollow, yellow to slightly greenish-yellow, smooth, sticky or glutinous with a layer of viscid and translucent material when wet, nearly equal mostly but usually tapering at base.

Basidiospores 7–9(11) × 5–6.5(7.5) μm, Q=1.25–1.7, Qm=1.48, ellipsoid, not constricted, smooth, hyaline, thin-walled. Basidia 29–39 × 7.5–10 μm, clavate, 4-spored; sterigmata up to 5 μm. Lamellar trama subregular, with hyphal elements 10–20 μm wide. Pileipellis an ixotrichoderm. Clamp connections present.

##### Habitat and known distribution.

Solitary or scattered, on moist ground in a mixed forest with mosses in North-eastern China, so far known in North America and East Asia.

##### Material examined.

CHINA. Jilin Province, Antu County, Changbaishan Mountains, 20 August 2012, Ming Zhang, Jiang Xu, Chao-Qun Wang (GDGM41710, GDGM42150 and GDGM42151).

**Figure 5.**
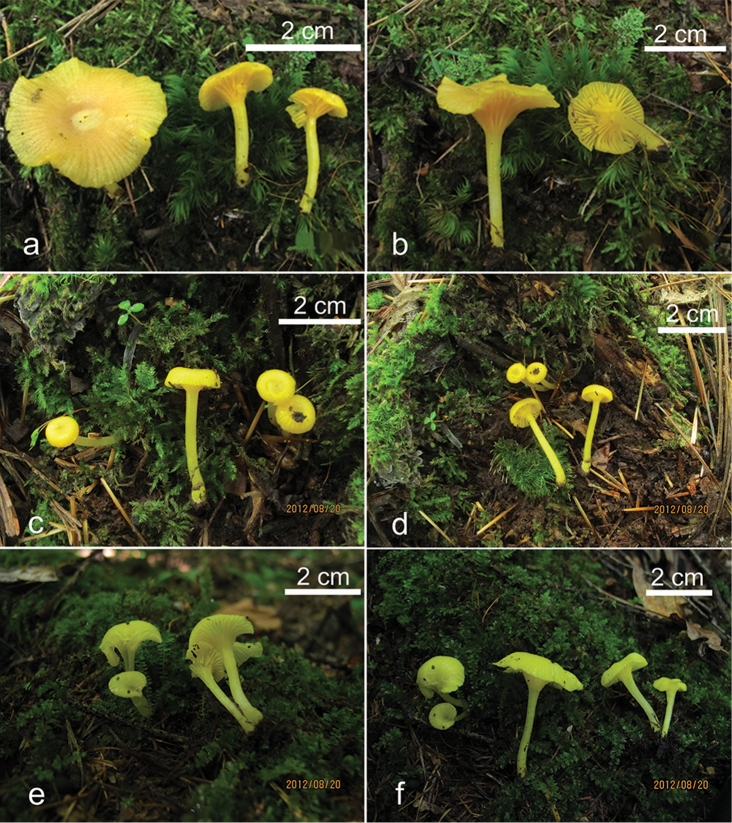
Basidiomata of *Gloioxanthomycesnitidus* (**a–b** GDGM41710 **c–d** GDGM42150 **e–f** GDGM42151).

**Figure 6.**
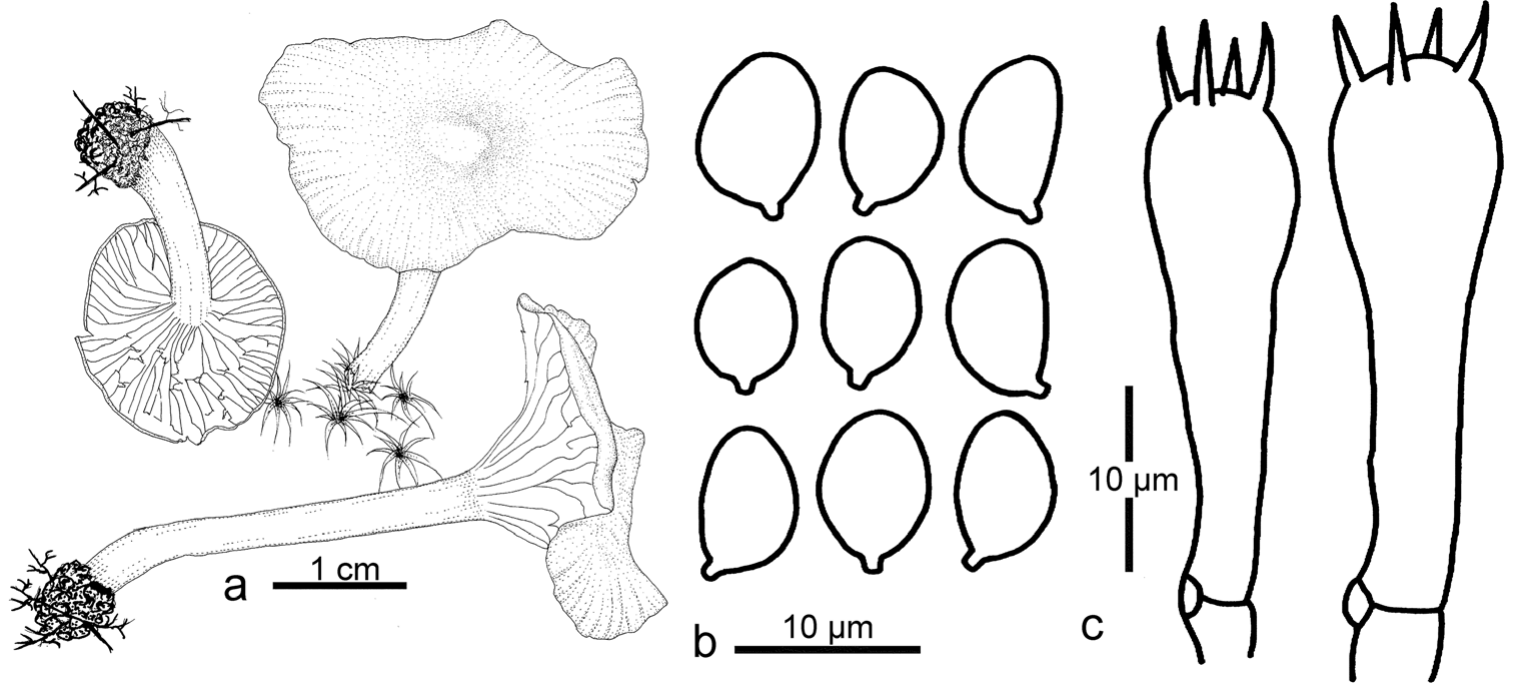
Line drawings of *Gloioxanthomycesnitidus* (GDGM41710). **a** basidiomata **b** basidiospores **c** basidia.

## Discussion

Phylogenetically, the distinction of the three subfamilies (Lodge et al. 2014) within Hygrophoraceae has very convincing support in the multi-locus tree of this study (Fig. 1). In addition, the establishment of the three well-defined monophyletic tribes in subfamily Hygrocyboideae is supported in this phylogenetic frame where the tribe Hygrocybeae with 73% support values and the tribe Humidicuteae with low support value are sister clades, while the tribe Chromosereae with 76% support values is located at their base. However, the cuphophylloid grade appears not to be monophyletic, thus more studies are still needed to understand the phylogenetic positions of *Ampulloclitocybe*, *Cantharocybe* H.E. Bigelow & A.H. Sm. and *Cuphophyllus* (Donk) Bon.

In the multi-gene analyses, *Sinohygrocybe* is placed together with two other genera in Chromoserae. *Chromosera* and *Gloioxanthomyces* are sister genera under the monophyletic tribe Chromosereae, while *Sinohygrocybe* is an independent generic lineage; and the distances between *Sinohygrocybe* and *Chromosera* or *Gloioxanthomyces* are further than the distance between *Chromosera* and *Gloioxanthomyces*. Such results are confirmed in the ITS phylogenetic tree (Fig. 2). According to the Blastn results, the ITS and LSU sequence identities of the new species to the known taxa are not more than 76% and 96%, respectively, with the *Chromosera* and *Gloioxanthomyces* sequences in GenBank. Thus, it is clear the new genus is independent of those two existed genera.

Beside the molecular analyses, morphological data also support its recognition within tribe Chromosereae. *Sinohygrocybe* shares a bright pileus colour and decurrent lamellae with the other genera *Chromosera* and *Gloioxanthomyces* (Table 2). However, the genus *Chromosera*, typified by *C.cyanophylla* (Fr.) Redhead, Ammirati & Norvell, differs from *Sinohygrocybe* in having omphaloid basidiomata, ephemeral dextrinoid reactions in the context, ratio of basidiospore to basidium length <5, ephemeral pigment bodies in the pileipellis and lilac pigments sometimes present (Redhead et al. 1995, Candusso 1997, Lodge et al. 2014); while *Gloioxanthomyces* differs from *Sinohygrocybe* by having weaker/delicate basidiomata, viscid pileus and stipe surface, gelatinised lamellar edge and cheilocystidia, shorter basidia (Boertmann 1990, 2012) with a length ratio of basidium to basidiospore 4–5. *Sinohygrocybe* shares some macroscopic characters with *Hygrocybe*, typified by *H.conica*, including bright colour of basidiomata and the distant lamellae, but *Hygrocybe* differs from *Sinohygrocybe* by having more fragile lamellae, more glabrous stipe (at least at the upper portion), often constricted spores and shorter basidia.

*Sinohygrocybe* samples were collected in both late summer (August) and winter (November), showing that they likely have a quite long fruiting season. It should be noted, however, that they are more abundant at times with lower temperature and higher humidity. Therefore, their fruiting in summer may occur only at higher altitude (with the elevation above 1500 m).

As to the Chinese new *Gloioxanthomycesnitidus* record: 1) phylogenetically, the Chinese samples are nested in the *Gloioxanthomyces* clade as a sister branch to the North American branch (Fig. 2); 2) morphologically, it shares these characters with the North American *G.nitidus*: deep yellow basidiomata fading to whitish with age, viscid, hygrophanous surface, central concave pileus and decurrent lamellae (Bessette et al. 2012); 3) geographically, *G.nitidus* and *G.vitellinus* are distributed in North America and Asia and Europe, respectively, indicating that *Gloioxanthomyces* is a Holarctic genus. It is assumed that both North American and East Asian *G.nitidus* were separated from the same ancestor because of geographical isolation, thus they are very similar at present; however, they may continue to diverge, eventually becoming separate species in the future since they live on detached continents.

**Table 2.** Type location, basidiospores and basidia dimensions of species of the tribe Chromosereae.

Species name	Type location	Basidiospores (μm)	Basidia (μm)	Reference
* Gloioxanthomyces nitidus *	USA, South Carolina	6.5–9(11) × 4–6.5(7.5)	29–39 × 7.5–10	Bessette et al. 2010, this study
* Gloioxanthomyces vitellinus *	Sweden	(6.5)7–9(9) × (5)5.5–7(7.5)	30–45 × 7–10	Boertmann 2010
* Chromosera citrinopallida *	USA, Washington	7–9(10) × 4.5–5	10–45 × 6–8	Smith and Hesler 1954
* Chromosera cyanophylla *	Sweden	(6.8)7.2–8.0(8.8) × (3.2)3.6–4.4	24–28 × 5.5–6.5	Holec et al. 2015
* Chromosera lilacina *	northern Fennoscandia	7–8.5(10) × (4)5–6(6.5)	30–45 × 7–9	Candusso 1997
* Chromosera viola *	Belgium, Namur Province	6.5–10.5(11) × 5–7(7.5)	36–61 × 8–11	Candusso 1997
* Chromosera xanthochroa *	Scotland	(5.5)6–8.5(10) × (3.8)4–5.2(5.5)	25–32 × 6.5–7.5(8.5)	Candusso 1997
* Sinohygrocybe tomentosipes *	China, Sichuan & Hunan Province	8–10(10.5) × (4.5)5–7(7.5)	41–80 × 4–10	This study

## Supplementary Material

XML Treatment for Sinohygrocybe

XML Treatment for Sinohygrocybe tomentosipes

XML Treatment for Gloioxanthomyces nitidus
